# Toll-Like Receptor-1 Single-Nucleotide Polymorphism 1805T/G Is Associated With Predisposition to Multibacillary Tuberculosis

**DOI:** 10.3389/fimmu.2018.01455

**Published:** 2018-06-25

**Authors:** Raphaela Honorato Barletta-Naveca, Felipe Gomes Naveca, Vanessa Alves de Almeida, Jorge Ivan Rebelo Porto, George Allan Villarouco da Silva, Mauricio Morishi Ogusku, Aya Sadahiro, Rajendranath Ramasawmy, Antonio Luiz Boechat

**Affiliations:** ^1^Laboratório de Micobacteriologia, Instituto Nacional de Pesquisas da Amazônia (INPA), Manaus, Brazil; ^2^Programa de Pós-graduação em Genética, Conservação e Biologia Evolutiva (PPG-GCBEv), Instituto Nacional de Pesquisas da Amazônia (INPA), Manaus, Brazil; ^3^Laboratório de Ecologia de Doenças Transmissíveis na Amazônia, Instituto Leônidas e Maria Deane, Fiocruz Amazônia, Manaus, Brazil; ^4^Programa de Pós-Graduação em Biologia da Interação Patógeno-Hospedeiro, Instituto Leônidas e Maria Deane, Fiocruz Amazônia, Manaus, Brazil; ^5^Programa de Pós-Graduação em Imunologia Básica e Aplicada, Universidade Federal do Amazonas, Manaus, Brazil; ^6^Programa de Pós-Graduação em Biologia Celular e Molecular, Instituto Oswaldo Cruz, Rio de Janeiro, Brazil; ^7^Laboratório de Genética Animal, Instituto Nacional de Pesquisas da Amazônia (INPA/CPBA), Manaus, Brazil; ^8^Laboratório de Imunologia Molecular, Instituto de Ciências Biológicas, Universidade Federal do Amazonas, Manaus, Brazil; ^9^Faculdade de Medicina, Universidade Nilton Lins, Manaus, Brazil; ^10^Fundação de Medicina Tropical Doutor Heitor Vieira Dourado (FMT-HVD), Manaus, Brazil; ^11^Laboratório de Imunoquímica, Instituto de Ciências Biológicas, Universidade Federal do Amazonas, Manaus, Brazil

**Keywords:** tuberculosis, immunogenetics, single-nucleotide polymorphism, toll-like receptors, Brazil

## Abstract

Tuberculosis (TB), caused by mycobacterial species of the *Mycobacterium tuberculosis* complex, is a serious global health issue. Brazil is among the 22 countries with the highest number of TB cases, and the state of Amazonas has the highest incidence of TB cases in the country. Toll-like receptors (TLRs) are important pattern recognition receptors of the innate immunity and play a key role in orchestrating an effective immune response. We investigated whether the single-nucleotide polymorphisms (SNPs) 1805T/G *TLR1*, 2258G/A *TLR2*, 896A/G and 1196C/T of *TLR4*, 745T/C *TLR6*, and −1237A/G and −1486A/G of *TLR9* are associated with the predisposition to TB and/or bacillary load. The SNPs genotyping was performed by nucleotide sequencing in 263 TB patients and 232 healthy controls residing in the state of Amazonas. Alleles and genotypes frequencies were similar between patients and healthy individuals for most of the investigated SNPs. Stratification of the TB patients according to their bacillary load showed that the genotype 1805TT *TLR1* (rs5743618) was prevalent among paucibacillary patients [odds ratio (OR) = 0.38; 95% confidence interval (CI) = 0.19–0.76; *p* = 0.009] while the genotype 1805TG was common among multibacillary patients (OR = 3.72; CI = 1.65–8.4; *p* = 0.004). Comparison of demographic characteristics of patients to controls showed that TB is strongly associated with smoking (OR = 6.55; 95% CI = 3.2–13.6; *p* < 0.0001); alcohol use disorder (OR = 7.14; 95% CI = 3.7–13.9; *p* < 0.0001); and male gender (OR = 3.66; 95% CI = 2.52–5.3; *p* < 0.0001). Multivariate logistic regression demonstrated that alcoholism (OR = 2.93; 95% CI = 1.05–8.16; *p* = 0.03) and the 1805G allele (OR = 2.75; 95% CI = 1.33–5.7; *p* = 0.006) are predictive variables for multibacillary TB. Altogether, we suggest that the *TLR1* 1805G allele may be a relevant immunogenetic factor for the epidemiology of TB together with environmental, sociodemographic, and behavioral factors.

## Introduction

Tuberculosis (TB) is a contagious disease caused by mycobacteria of the *Mycobacterium tuberculosis* complex. The most common clinical form is pulmonary TB. TB causes a serious public health burden worldwide and its control remains a major challenge for the scientific community and TB control programs globally. Nearly 10 million people worldwide suffered from TB each year (1). Brazil is one of the 30 countries considered a priority for TB control as more than 69 thousand new cases occur annually. The state of Amazonas has the highest incidence of TB in the country with 67.2/100 thousand inhabitants (1, 2).

Multiple socioeconomic, behavioral, medical, and genetic factors affect TB infection (3, 4). Many single-nucleotide polymorphisms (SNPs) in immune response genes are associated with TB (5–7). The most reported are *HLA-DRB1, VDR, TIRAP*, and *NRAMP1* (6, 8). Other genes such as toll-like receptors (TLRs), *TLR1*, 2, 4, 6, and 9 that are involved in the initial recognition of pathogen-associated molecular patterns to trigger the activation of the effector mechanisms of innate immunity and subsequent targeting of the specific adaptive immune response to *Mycobacterium tuberculosis* have also been associated with susceptibility or resistance to TB (6, 9, 10).

In this study, we investigated whether the SNPs rs5743618 (1805T/G) of *TLR1*, rs5743708 (2258G/A) of *TLR2*, rs4986790 (896A/G) and rs4986791 (1196C/T) of *TLR4*, rs5743810 (745C/T) of *TLR6*, and the rs5743836 (−1237A/G) and rs187084 (−1486A/G) of *TLR9* are associated with TB and with the bacterial load. We identified that alcoholism and the *TLR1* 1805G allele may be predictive variables for multibacillary TB.

## Materials and Methods

### Study Population

This is a case-control study investigating the association of demographic and immunogenetics factors with susceptibility or resistance to TB. Patients with TB and healthy controls, aged 18–65 years, were recruited in Manaus, the capital city of Amazonas state, Brazil. The patients with TB were recruited at the Policlínica de Referência em Pneumologia Sanitária Cardoso Fontes, a referral center of the state for diagnosis and treatment of TB. Patients with a diagnosis of pulmonary and pleural TB were selected according to guidelines of the Brazilian Ministry of Health. Data collected included social-demographic characteristics and the presence of the following clinical symptoms: cough, expectoration, chest pain, dyspnea, hemoptysis, malaise, fever, chills, night sweats, loss of appetite, weight loss, and fatigue. The controls consisted of health professionals and healthy contacts without consanguinity to the patients with TB and without any previous history of the disease. TB patients with associated comorbidities or lack of information on the patient’s chart or subsequent diagnosis of non-tuberculous mycobacteria were excluded. TB patients were stratified into paucibacillary (including pleural TB) and multibacillary. The bacillary load was determined by bacilloscopy, as recommended by Brazilian Ministry of Health (11). This study protocol was approved by the Institutional Review Board of the National Institute of Amazonian Research (INPA) under the number CAAE: 05925212.8.0000.0006.

### Identification of *M. tuberculosis*

Sputum samples were submitted to microscopy (12) and solid culture in according to PKO method (initials in tribute to Petroff, Kudoh, and Ogawa) (13). A smear of culture, stained by Kinyoun method, was carried out to confirm if the culture was composed of acid-fast bacilli. Later, the colony aspect (not pigmented and rough morphology) was evaluated. The DNA extraction from pure culture was made as described elsewhere (14). So, the molecular identification of *M. tuberculosis* complex was performed by PCR, amplification of 123 bp fragment from IS*6110* insertion sequence, as described (15). If, the PCR for IS*6110* was negative, the differential diagnosis of mycobacteria was performed as established (12, 16). This study also included samples (32/263) with diagnosis confirmed for TB by the molecular test, GeneXpert MTB/RIF [*M. tuberculosis*/rifampin (RIF) resistance] assay (Cepheid, Sunnyvale, CA, USA).

### Genotyping of the Different TLR Polymorphisms

DNA from whole blood samples was extracted using a QIAamp DNA Blood kit (QIAGEN) in the automated QIAcube platform following the manufacturer’s instructions. The primers were designed to flank the different polymorphisms studied using the Primer-3 version 2.3.7 embedded in Geneious software version 6.1.7 (17) and Primer-BLAST (Table S1 in Supplementary Material). PCR conditions for the five evaluated genes were optimized separately to use only one cycling amplification program of an initial cycle of 94°C for 2 min followed by 33 cycles of 94°C for 2 min, 63°C for 30 s and 72°C for 60 s, and a final cycle of 72°C for 17 min. The PCR mix in a final volume of 25 µL contains 1× Buffer; 1.5 mM MgCl_2_; 0.2 mM dNTPs; 0.4 µM of sense and antisense primers; enzyme GoTaq Hot Start 1 U/μL; and approximate 100 ng of DNA. PCR products were precipitated with polyethylene glycol (PEG) (PEG 8000 at 20% P/V, 2.5 M NaCl) based on the protocol originally described by Lis and Schleif (18), with slight modifications subsequently by Lis (19) and Paithankar and Prasad (20). The purified amplicons were adjusted to the concentration range of 5–20 ng/µL, proportional to their size. The allelic discrimination was performed by direct capillary sequencing. Sequencing reaction was performed with the BigDye^®^ Terminator Kit (3.1) (Life technologies) using the protocol suggested by Platt et al. (21). Either sense or antisense primers used in PCR of each gene was used. The sequencing product was purified with Ethanol/EDTA/Sodium Acetate, according to the recommendations of Life Technologies and submitted to capillary electrophoresis in the ABI 3130 Genetic Analyzer (Applied Biosystems) and the resulting electropherograms were edited and analyzed using the Sequencing Analysis Program (Life Technologies, version 5.3.1) and Geneious.

### Statistical Analysis

Parametric Student’s *t*-test was used for means age comparison. Two-tailed Fisher’s exact test was applied for association of TB with BCG vaccine, sex, smoking, and alcoholism. The frequency of the alleles and genotypes between groups was compared with two-tailed Fisher’s exact test. Variables with a *p* value were considered significant. Genetic models also were applied to elucidate the association of the SNPs with susceptibility to TB and bacillary load. Akaike information criterion (AIC) was considered in the evaluation of applied genetic models. The free software R, version 3.1.2 and the embedded function to study SNP association—SNPassoc^1^ were used. The multivariate logistic regression analysis with stepwise approach was applied in the choice of variables predictive of multibacillary TB. The analyzed variables were age, sex, BCG, alcoholism, and the presence of the 1805G allele. Variables with a *p* value <0.05 were considered and the variables *p* >0.1 were excluded from the regression analysis. For each regression model proposed, the discrimination capacity of each model was analyzed as described previously by Boechat et al. (22, 23). The model with the largest area under the curve (AUC) was considered. Statistical analyses were performed using MedCalc Statistical Software version for Windows, version 15.2 (MedCalc Software, Ostend, Belgium^2^).

## Results

### Baseline Characteristics of All the Participants of the Study

This study included 263 patients with TB and 232 healthy controls. The baseline characteristics of the study population are shown in Table 1. The age distribution between TB patients and controls was similar. TB patients had a mean of 33.4 ± 11.9 years of age and were predominantly male (65.7%). By contrast, the control group was predominantly female (65.9%). BCG vaccine adherence was higher among the controls, 92.0% compared to 78.7% among the TB patients. Smoking and alcoholism were significantly associated with a greater predisposition to TB. The clinical characteristics of the patients with TB are shown in Table 2. Pulmonary TB (88%) was the most prevalent and the majority of the patients reported more than three clinical symptoms.

**Table 1:** Baseline characteristics of patients with TB and the healthy controls.

General characteristics	TB patients (*n* = 263)	Controls (*n* = 232)	OR	95% CI	*p-*Value
Age	33.4 ± 11.9	33.5 ± 11.9	–	–	0.92
Male (%)	172 (65.4)	79 (34.1)	3.66	2.52–5.3	<0.0001
Vaccine BCG (%)	207 (78.7)	213 (92)	0.26	0.13–0.5	<0.0001
Smoking (%)	55 (20.9)	9 (3.9)	6.55	3.2–13.6	<0.0001
Alcohol use disorder (%)	69 (26.2)	11 (4.7)	7.14	3.7–13.9	<0.0001

*TB, tuberculosis*.

**Table 2:** Clinical characteristics of the patients with TB.

Characteristics	Patients (*n* = 263) *n*(%)
**Clinical form of TB**	
Pulmonary	231 (88.0)
Pleural	32 (12.0)

Family history of TB	136 (51.7)
**Bacillary load^a^**	
Multibacillary	176 (76.2)
Paucibacillary	55 (23.8)
**Clinical symptoms**	
Coughing with 1 symptom	19 (7.2)
Coughing with 2 symptoms	23 (8.8)
Coughing with 3 symptoms	35 (13.3)
Coughing with 4 or more symptoms	172 (65.4)
No information	14 (5.3)

*All patients with pleural TB were grouped as paucibacillary*.

*^a^Bacillary loads for 32 patients were not available but were confirmed for Mycobacterium tuberculosis by Xpert MTB/RIF*.

*TB, tuberculosis*.

*Clinical symptoms in addition to the cough: expectoration, chest pain, dyspnea, hemoptysis, malaise, fever, chills, night sweats, loss of appetite, weight loss, and fatigue*.

### Comparison of the Genotypes and Alleles Frequencies of the Polymorphisms of the Different TLRs Between TB Patients and Healthy Controls

The genotypes and alleles frequencies of the different SNPs studied are shown in Table 3. Differences in the number of samples for each SNP are due to the rigorous adoption of only high-quality sequences analyzed. The Hardy–Weinberg equilibrium (HWE) was observed for the different SNPs in both cases and healthy controls except for both SNPs of *TLR4*. Further analysis of the SNPs of *TLR4* was abandoned due the deviations from the HWE. The frequencies of the genotypes and alleles of the other SNPs were similar between patients and controls except for the SNPs of *TLR9*. Notably, *TLR2* SNP 2258G/A was not observed in our population of study. *TLR9* −1237A/G and −1486A/G SNPs showed minor allele frequency (MAF) for patient populations and controls of 15.3 and 17.4% for the first SNP and 38.5 and 33.3% for the second, respectively. The genotype −1237AG was associated with TB protection [odds ratio (OR): 0.62; confidence interval (CI): 0.39–0.97; *p* = 0.04].

**Table 3:** Genotypes and allele frequencies of the TLRs polymorphisms in healthy controls and patients with TB.

	Controls *n*(%)	Patients with TB *n*(%)	OR	CI 95%	*p*-Value
Genotypes					

***TLR1*** 1805T/G (rs5743618)					

T/T	116 (55.3)	146 (58.0)	1.11	0.7–1.61	
T/G	74 (35.2)	86 (34.1)	0.64	0.64–1.37	0.77
G/G	20 (9.5)	20 (7.9)	0.81	0.42–1.56	
Alleles					
T	306 (73.0)	378 (75.0)	1.11	0.83–1.5	0.48
G	114 (27.0)	126 (25.0)	0.89	0.66–1.37	

***TLR2*** 2258G/A (rs5743708)					

G/G	168 (100)	196 (100)	–	–	
G/A	0	0	–	–	–
A/A	0	0	–	–	
Alleles					
G	336(100)	392 (100)	–	–	–
A	0	0	–	–	

***TLR4*** 896A/G (rs4986790)					

A/A	199 (95.7)	221 (92.9)	0.5	(0.2–1.3)	0.41
A/G	8 (3.8)	16 (6.7)	1.8	(0.7–4.3)	
G/G	1(0.5)	1 (0.4)	–	–	
Alleles					
A	406 (97.6)	458 (96.2)	0.62	(0.29–1.37)	0.20
G	10 (2.4)	18 (3.8)	1.6	(0.73–3.5)	

***TLR4*** 1196C/T (rs4986791)					

C/C	207 (99.5)	226 (95)	0.75	(0.3–1.88)	0.17
C/T	1 (0.5)	11 (4.6)	1.39	(0.52–3.65)	
T/T	0	1 (0.4)			
Alleles					
C	415 (99.8)	463 (97.3)	0.79	(0.33–1.86)	0.003
T	1 (0.2)	13 (2.7)	1.27	(0.54–3.0)	

***TLR6*** 745T/C (rs5743810)					

T/T	120 (69)	176 (73)	1.2	(0.7–1.8)	
T/C	50 (29)	58 (24)	0.7	(0.5–1.2)	0.47
C/C	4 (2)	8 (3)	1.45	(0.43–4.9)	
Alleles					
T	290 (83)	410 (84.7)	1.1	(0.76–1.61)	0.6
C	58 (17)	74 (15.3)	0.9	(0.9–1.3)	

***TLR9*** −1237A/G (rs5743836)					

A/A	127 (66)	141 (73)	1.38	(0.89–2.14)	
A/G	63 (33)	45 (23)	0.62	(0.39–0.97)	0.04
G/G	2 (1)	7 (4)	3.75	(0.73–17.4)	
Alleles					
A	317 (82.6)	327 (84.7)	1.17	(0.79–1.71)	0.42
G	67 (17.4)	59 (15.3)	0.85	(0.58–1.25)	

***TLR9*** −1486A/G (rs187084)					

A/A	84 (44)	67 (34.9)	0.65	(0.43–0.98)	
A/G	88 (46)	102 (53.1)	1.33	(0.89–2)	0.21
G/G	20 (10)	23 (12)	1.17	(0.61–2.21)	
Alleles					
A	256 (66.7)	236 (61.5)	0.79	(0.59–1.07)	0.13
G	128 (33.3)	148 (38.5)	1.25	(0.93–1.68)	

*Total of controls (C) and patients (P) analyzed for each SNP: TLR1 1805T/G 210 (C) 252 (P); TLR2 2258G/A 168 (C) 196 (P); TLR4 896A/G 208 (C) 238 (P); TLR4 1196A/G 208 (C) 238 (P); TLR6 745T/C 174 (C) 242 (P); TLR9 −1234 A/G 192 (C) 193 (P); TLR9 −1486 A/G 192 (C) 192 (P)*.

*CI, confidence interval; OR, odds ratio; TLRs, toll-like receptors; TB, tuberculosis; SNP, single-nucleotide polymorphism*.

### Association of *TLR1* SNP 1805T/G and Multibacillary TB

Patients with TB were stratified into paucibacillary and multibacillary TB. Comparison of genotypes and allele frequencies of all the SNPs between multibacillary and paucibacillary TB revealed that the genotype 1805TT *TLR1* (rs5743618) was prevalent among paucibacillary patients (OR = 0.38; 95% CI = 0.19–0.76; *p* = 0.009) while the genotype 1805TG was common among multibacillary patients (OR = 3.72; CI = 1.65–8.4; *p* = 0.004). The 1805G allele (OR = 1.71; CI = 0.97–3.01; *p* = 0.05) showed statistical trend for association. Further analysis applying four genetic models as shown in Table 4 showed that the codominant model with the genotype (1805TG) was associated with the multibacillary TB form (OR = 3.69; CI = 1.62–8.42; *p* = 0.002). The same genotype was associated with the multibacillary form in the overdominant model (OR = 3.73; CI = 1.65–8.41; *p* = 0.0004). In the dominant model, the genotypes (1805TG + 1805GG) also showed association with the multibacillary TB (OR = 2.63; CI = 1.31–5.29; *p* = 0.004). The overdominant model better explains the association to multibacillary TB according to AICs results.

**Table 4:** Different genetic models of association of *TLR1* SNP with clinical forms of TB.

Model	PB *n*(%)	MB *n*(%)	OR	95% CI	*p*-Value	AIC
**Codominant**						
T/T	38 (74.5)	90 (52.6)	1.00	–	0.002	233.2
T/G	8 (15.7)	70 (40.9)	3.69	1.62–8.42		
G/G	5 (9.8)	11 (6.5)	0.93	0.30–2.86		

**Dominant**						
T/T	38 (74.5)	90 (52.6)	1.00	–	0.004	235.2
T/G—G/G	13 (25.5)	81 (47.4)	2.63	1.31–5.29		

**Recessive**						
T/T—T/G	46 (90.2)	160 (93.6)	1.00	–	0.429	242.7
G/G	5 (9.8)	11 (6.4)	0.63	0.21–1.91		

**Overdominant**						
T/T—G/G	43 (84.3)	101 (59.1)	1.00	–	0.0004	231.2
T/G	8 (15.7)	70 (40.9)	3.73	1.65–8.41		

**Log-additive**						
0, 1, 2	51 (23.0)	171 (77.0)	1.68	0.96–2.92	0.057	239.7

*Of note, the genotypes of 30 TB patients out of 252 sequenced for TLR1 were excluded since only results of GeneXpert MTB/RIF without bacilloscopy were available*.

*PB, paucibacilary; MB, multibacilary; CI, confidence interval; OR, odds ratio; AIC, Akaike information criterion; TLRs, toll-like receptors; SNP, single-nucleotide polymorphism; TB, tuberculosis*.

### Alcoholism and the *TLR1* SNP Guide Predisposition Multibacillary TB

Multivariate logistic regression analysis was applied to adjust baseline characteristics, using a logistic regression model for multibacillary TB shown in Table 5, including gender, age, smoking status, alcoholism, and citizenship. There was no influence for gender in the analysis (*p* = 0.96). Logistic regression showed the variables alcoholism (OR = 2.93; CI = 1.05–8.16; *p* = 0.03) and allele 1805G (OR = 2.75; CI = 1.33–5.7; *p* = 0.006) were associated with the multibacillary TB (Table 5).

**Table 5:** Multivariate logistic regression for multibacillary TB.

Variables	OR	95% CI	*p*-Value
Age	0.98	0.96–1.01	0.29
Male	0.98	0.5–1.95	0.96
Smoking	1.18	0.42–3.35	0.74
BCG	0.71	0.3–1.67	0.43
Alcoholism	2.93	1.05–8.16	0.03
Citizenship	1.12	0.58–2.2	0.72
1805G	2.75	1.33–5.7	0.006

*CI, confidence interval; OR, odds ratio; TB, tuberculosis*.

Logistic regression with a stepwise approach only showed alcoholism (OR = 3.04; CI = 1.21–7.67; *p* = 0.01) and allele 1805G (OR = 2.59; CI = 1.28–5.26; *p* = 0.008) were associated with the multibacillary TB. The two variables were subsequently challenged in two regression models. Model 1 that included only the variable alcoholism, obtained an AUC of 0.58. In the model 2 that included both the variables alcoholism and SNP 1805T/G, the AUC increased to 0.64 suggesting that both alcoholism and SNP 1805T/G act together in the establishment of multibacillary TB.

## Discussion

Tuberculosis continues to be a health problem worldwide and with the emergence of HIV there is a resurgence of TB cases among AIDS patients and becomes barriers to the roll back of TB. Enormous headway has been achieved in the understanding of the immunopathology of TB. Understanding the puzzle of the host genetics contribution to the resistance or susceptibility to MTB infection may help in the strategy of designing new vaccines to TB or in the combination of chemotherapy with immunotherapy.

None of the SNPs investigated in this study were associated with a greater predisposition to TB. Only the *TLR9* −1237AG genotype was associated with TB protection. Stratification of the patients into multibacillary and paucibacillary TB did not show any association with the bacillary load. Few studies evaluated the importance of SNPs −1237A/G and −1486A/G of *TLR9* in TB, and results are conflicting. Two cited no association with TB (24, 25) while two reported association with higher risk for TB (26, 27). The predisposition to TB is known to be complex and is determined by many genetic variations in a multiple of genes contributing to smaller inputs in the development of the disease (5, 10).

We observed the 1805TG genotype is associated for the first time to the best of our knowledge with multibacillary TB. Interestingly, it has been shown that *TLR1* with this variation is hyporesponsive to its agonists and impedes its migration to the surface of cells expressing it (28–30). Among the demographic and behavioral factors investigated, only alcoholism contributed to the multibacillary form.

A previous study reported that *TLR1* regulates polypeptide-induced signaling and responds to extracts of *M. tuberculosis*, suggesting that *TLR1* may substantially regulate the immune response against the bacillus and impact on the outcome of TB. Authors also demonstrated that subjects with *TLR1* SNP1805G/G genotype produce low concentrations of IL-6, an important cytokine in the inflammatory response. Heterozygous individuals produce intermediate levels suggesting that the G and T allele are codominant. The association of heterozygous individuals to multibacillary TB in this study may in part be explained by the influence of the G allele that correlates to low level of IL-6 to inefficiently control the multiplication of the bacillus (29). However, the effect of the hypo-responsiveness of this variation on the risk of TB and the bacillary load is not clearly understood due to the complex immune response to TB and the immune escape developed by *M. tuberculosis* (31, 32).

In this study, we observed that males, smokers, and alcoholism were more common among the TB patients. Furthermore, family history of TB was reported in more than 50% of the patients reinforcing the importance of intra-domiciliary TB transmission or the component of genetic factors in the predisposition to TB. Previous studies have also observed similar features and reinforced that smoking, alcohol, and other drug abuse, along with socioeconomic factors, increase the risk for TB development (33).

There is biological evidence to explain the association of smoking with TB. Chronic exposure to cigarette components alters the normal functioning of mucociliary clearance. This facilitates the access of the bacilli to the pulmonary alveoli, giving rise to an infectious focus. Macrophages resident in the alveola of smokers also present reduced phagocytic action and decreased level of pro-inflammatory cytokines. Nicotine, one of many toxic constituents found in cigarettes, acts directly on acetylcholine receptors in macrophages and leads to a lower secretion of TNF-alpha, generating a favorable environment for bacillary survival in the macrophages and contributes to immune evasion (34, 35).

Variations in the frequency of SNPs suggest distinct evolutionary histories among populations in recent human evolution due to selective pressure exerted by diseases, such as TB and Malaria (36). This study observed MAF for SNP 1805T/G of *TLR1* of 25.0 and 27.0% in TB patients and controls, respectively. Previous studies of other infectious diseases in Brazil showed frequencies varying between 11.0 and 40.0% (37–40). Frequency of the MAF in other populations such as Spain, Turkey, and Nepal are 30.0, 43.0, and 6.0%, respectively (28, 41, 42). The 1805G mutant allele is rare in Vietnamese population (28).

*TLR2* SNP 2258G/A was not observed in our study. This SNP is also rare in four populations investigated by the HapMap project and in a population from Colombia (43). The *TLR4* 896A/G and 1196C/T SNPs are co-segregated and the frequency of haplotype of the mutant alleles ranges from 0 to 5.0% among different populations worldwide. However, this frequency is 10–20 times higher among African populations and it is suggested that may be a result of selective pressure due to malaria infection (44, 45). Our study observed an MAF of 3.8 and 2.4% for 896A/G, and 2.7 and 0.2% for the 1196C/T SNP among patients and controls, respectively. Other studies in Brazil showed higher MAF varying from 4.6 to 7.0% (37, 38, 46). However, one study of case-controls of malaria from the Amazonas observed similar MAF to ours indicating the frequency is a reflex of the local population (40).

The MAF of *TLR6* 745C/T varies from 0 to 46.0% among 11 populations investigated by the HapMap. In this study, the MAF is 15.3 and 17% in TB patients and controls, respectively. Another study in Brazil reported an MAF of 18.0 and 10.0% in individuals of symptomatic and asymptomatic malaria, respectively (37). The MAF of *TLR9* −1237A/G and −1486A/G SNPs are 15.3 and 17.4% for the first SNP and 38.5 and 33.3% for the second in patients and controls, respectively. Similar high MAF for these SNPs were observed in other studies in the Amazonas (37, 40).

Our study has few limitations. Our sample size is small, and male is prevalent among the TB cases. However, this should not have a great influence on the study as the frequencies of the alleles of males and females are similar among the controls. Moreover, new study with a higher sample size is needed to confirm the results observed in this study.

The predisposition to TB and its bacillary load involves numerous factors that are determined mainly by the human intrinsic factors and the virulence of the mycobacterial strain. Further studies are necessary to evaluate the combined effect of these factors in TB. In addition, more studies are needed to understand the functional effect of this *TLR1* variant to elucidate its importance in the regulation of the immune response and in the immunopathogenesis of TB.

## Ethics Statement

This study protocol was approved by the Institutional Review Board of the Instituto Nacional de Pesquisas da Amazônia (INPA) under the number CAAE: 05925212.8.0000.0006. All patients gave written informed consent.

## Author Contributions

RB-N, FN, AS, AB, RR, and JP conceived and designed the study. RB-N, VA, MO, and AS were responsible for acquisition and quality of the clinical and laboratory data. RB-N, VA, AS, and MO performed the identification of *Mycobacterium tuberculosis*. RB-N, FN, and GS performed the genotyping of the different TR polymorphisms. RB-N, FN, and AB conducted data mining and statistical analysis. RB-N, FN, MO, AS, AB, and RR wrote the first version of the paper. All authors were involved in the interpretation of the data and participated in the final writing of the manuscript.

## Conflict of Interest Statement

The authors declare that the research was conducted in the absence of any commercial or financial relationships that could be construed as a potential conflict of interest. The reviewer MS and handling Editor declared their shared affiliation.

## Footnotes

^1^https://cran.r-project.org/web/packages/SNPassoc/index.html (Accessed: March 09, 2016).

^2^https://www.medcalc.org/ (Accessed: January 18, 2016).
