# Supplementary material for: Toll-Like Receptor-1 Single-Nucleotide Polymorphism 1805T/G Is Associated With Predisposition to Multibacillary Tuberculosis
Source: Front Immunol. 2018 Jun 25;9:1455. doi: 10.3389/fimmu.2018.01455 (PMC6026633; doi:10.3389/fimmu.2018.01455)
Supplement: Supplementary file 1 [file Table_1.docx]

**Supplementary Table 1. Toll-like Receptors primers used in this study.**

| **Gene / chromosome** | **Primer name** | **Primer sequence (5’– 3’)** | **Region of interest** | **Target position** | **Tm ^o^C** | **GC%** | **Amplicon size (bp)** |
| --- | --- | --- | --- | --- | --- | --- | --- |
| ***TLR1***  **4p14**  * NG_016228.1 | TLR1_RNF | ATGTTTACCTCCCAGGATCAAGGT | exon 4 | 12283 - 12306 | 65.3 | 45.8 | 631 |
|  | TLR1_RNR | CACCCAGAAAGAATCGTGCCCA |  | 12913 - 12892 | 66.7 | 54.5 |  |
| ***TLR2***  **4q31.3**  * NG_016229.1 | TLR2_RNF | CCTTCACTCAGGAGCAGCAAGCAC | exon 3 | 1884-1907 | 68.7 | 58.3 | 680 |
|  | TLR2_RNR | AGCCTCGTCCATGGGCCACT |  | 2563-2544 | 70 | 65 |  |
| ***TLR4***  **9q33.1**  * NG_011475.1 | TLR4_RNF | CCATCGTTTGGTTCTGGGAGA | exon 3 | 13712 - 13732 | 64 | 52.4 | 584 |
|  | TLR4_RNR | TGAGGTTTCTGAGTGATAGGAA |  | 14295 - 14274 | 60.7 | 40.9 |  |
| ***TLR6***  **4p14**  * NG_028087.1 | TLR6_RNF | GGCCCTGCCCATCTGTAAGGA | exon 2 | 32746 - 32766 | 67.9 | 61.9 | 658 |
|  | TLR6_RNR | GCTTGGTGCATGAGGACACAGCAT |  | 33403 - 33380 | 69 | 54.2 |  |
| ***TLR9***  **3p21.2**  * NG_033933.1 | TLR9_RNF | TGTGCCTGCCCAGAGCTGAC | promoter | 4035-4054 | 68.7 | 65 | 866 |
|  | TLR9_RNR | CGCCAGCCAGTCCTCCCCAT |  | 4900 - 4881 | 70.4 | 70 |  |

Primers used to amplify the regions of interest in each TLR gene, as well as for nucleotide sequencing. * NCBI RefSeqGene used for primer design. Tm were calculated with OligoAnalyzer 3.1 Integrated DNA Technologies (IDT) available at <https://www.idtdna.com/calc/analyzer>
